# The detection of *GRN* mutation carriers by progranulin blood protein levels from finger‐stick collection

**DOI:** 10.1002/alz.14259

**Published:** 2024-11-30

**Authors:** Hanna Huber, Valentina Cantoni, Daniele Altomare, Lana Grötschel, Laia Montoliu‐Gaya, Francisco Meda, Hlin Kvartsberg, Ilenia Libri, Maria Sofia Cotelli, Henrik Zetterberg, Kaj Blennow, Barbara Borroni, Nicholas J. Ashton

**Affiliations:** ^1^ Department of Psychiatry and Neurochemistry Institute of Neuroscience & Physiology the Sahlgrenska Academy at the University of Gothenburg Mölndal Sweden; ^2^ Department of Clinical and Experimental Sciences University of Brescia Brescia Italy; ^3^ Neurology Unit Vallecamonica Hospital Esine Italy; ^4^ Clinical Neurochemistry Laboratory Sahlgrenska University Hospital Mölndal Sweden; ^5^ Department of Neurodegenerative Disease Institute of Neurology UCL London UK; ^6^ UK Dementia Research Institute UCL London UK; ^7^ Hong Kong Center for Neurodegenerative Diseases Shatin Hong Kong China; ^8^ Wisconsin Alzheimer's Disease Research Center University of Wisconsin School of Medicine and Public Health Madison Wisconsin USA; ^9^ Paris Brain Institute ICM Pitié‐Salpêtrière Hospital Sorbonne University Paris France; ^10^ Neurodegenerative Disorder Research Center Division of Life Sciences and Medicine and Department of Neurology Institute on Aging and Brain Disorders University of Science and Technology of China and First Affiliated Hospital of USTC Hefei Anhui P.R. China; ^11^ Institute of Psychiatry Psychology and Neuroscience Maurice Wohl Institute Clinical Neuroscience Institute London King's College London London UK; ^12^ Banner Sun Health Research Institute Sun City Arizona USA; ^13^ Banner Alzheimer's Institute Phoenix Arizona USA

**Keywords:** biomarker, blood, clinical trials, finger‐prick, frontotemporal dementia, granulin, mutation, progranulin

## Abstract

**INTRODUCTION:**

Heterozygous mutations in the progranulin gene (*GRN*) leading to decreased progranulin levels are one of the most frequent causes of inherited frontotemporal dementia (FTD). We evaluated progranulin levels in dried blood spots from capillary finger‐stick collection (DBS^capillary^).

**METHODS:**

Paired venous Ethylenediaminetetraacetic acid (EDTA) plasma and DBS^capillary^ samples were collected from each participant with or without pathogenic *GRN* mutations.

**RESULTS:**

DBS^capillary^ progranulin levels in *GRN* mutation carriers (mean [SD] age, 55 [13] years; *n* = 16) were reduced compared to non‐mutation carriers (64 [11] years; *n* = 44) (2.38 ng/mL [1.0] vs 4.37 [0.68] ng/mL; *U* = 42; *p *< 0.0001, ROC AUC = 0.94 [95% CI: 0.83 to 1.00]) and highly associated with venous plasma levels (*R* = 0.819; *p *< 0.001).

**DISCUSSION:**

Progranulin levels can be accurately determined from finger‐stick blood samples. This can enable regular and remote monitoring of this protein in FTD therapeutic trials and potentially serve as a first‐level screening test for GRN mutations.

**Highlights:**

- Progranulin levels measured using capillary dried blood spots were significantly reduced in *GRN* mutation carriers compared to non‐mutation carriers.
- Progranulin levels measured using capillary dried blood spots strongly correlated with levels from venous EDTA plasma.
- DBS^capillary^ progranulin levels were able to identify *GRN* mutation carriers with high accuracy.
- DBS^capillary^ might allow repeated measurements of progranulin levels in a remote and unsupervised setting, circumventing the restrictions of traditional venous blood collection.
- DBS^capillary^ might be used to assess the biological efficacy of disease‐modifying therapies in clinical trials aiming to increase baseline progranulin levels or as a first‐level screening for *GRN* mutations in primary settings.

## 1 INTRODUCTION

Heterozygous mutations in the progranulin gene (*GRN*) are one of the most frequent causes of inherited frontotemporal dementia (FTD), presenting with behavioral variant FTD, primary progressive aphasia, or corticobasal syndrome phenotypes.^1^ These pathogenic *GRN* variants cause haploinsufficiency, resulting in a significantly decreased concentration of the progranulin protein, which is detectable in blood.^2^ The reduction of progranulin is already detectable in the presymptomatic disease stages.^3^

The identification of this core pathophysiological process was associated with unprecedented advancements in therapeutic approaches in individuals with *GRN* mutations. Ongoing pharmacological trials with monoclonal antibodies, brain shuttle proteins, or gene therapy aim to rescue progranulin concentrations in mutation carriers.^4^, ^5^ However, current and future clinical trials need reliable and accessible tools to monitor progranulin changes as an outcome of target engagement and biological efficacy. In turn, new approaches must be developed to easily carry out repeated measurements of progranulin over time in *GRN* mutation carriers, which will circumvent the limitations of venous blood collection.

Recently developed extraction methods and analysis of dried plasma or blood spots from venous or finger‐stick (DBS^capillary^) collection^6^, ^7^ represent an exciting new avenue in the field in that such methods accurately detect blood biomarkers of neurodegenerative diseases. These methods, particularly DBS^capillary^, would allow simplified, less‐invasive, remote, and potentially unsupervised sample collection facilitating regular biological assessment of patients or participants in a range of disease‐modifying clinical trials.

The aim of this study was to assess whether progranulin levels could be quantified in DBS^capillary^, whether such levels correlated with venous plasma progranulin, andwhether progranulin levels from DBS^capillary^ could be used to accurately identify GRN mutation carriers.

## 2 MATERIAL AND METHODS

### 2.1 Participants

Sixty participants were enrolled at the Center for Neurodegenerative Disorders, University of Brescia (Italy), from October 3, 2023, to November 27, 2023. The participants met current clinical criteria for the diagnosis of FTD^8^, ^9^ (*n* = 21) or Alzheimer's disease (AD) (*n* = 10).^10^ In FTD patients, the presence (*n* = 5) or absence (*n* = 16) of pathogenetic *GRN* mutations was evaluated according to standard procedures.^11^ Presymptomatic *GRN* mutation carriers^12^ (*n* = 11) and healthy controls (HCs) (*n* = 18), recruited among patients’ spouses or family members, were also included. Demographic and clinical characteristics are reported in Table 1. All participants gave written informed consent according to the Declaration of Helsinki. The study was approved by the local ethics committee (NP 2189).

**TABLE 1 alz14259-tbl-0001:** Participants’ demographic and clinical features.

	*GRN* mutation carriers			*GRN* non‐mutation carriers				
Variables	All *n* = 16	FTD *n* = 5	Presymptomatic *n* = 11	All *n* = 44	FTD *n* = 16	AD *n* = 10	HC *n* = 18	*p* value^*^
Age, years	55 (13)	61 (11)	52 (14)	64 (11)	68 (10)	66 (10)	60 (11)	0.006
Sex, female% (*n*)	75 (12)	80 (4)	73 (8)	48 (21)	19 (3)	60 (6)	67 (12)	0.113^a^
Education, years	12 (3)	13 (3)	12 (3)	11 (4)	11 (5)	11 (4)	11 (4)	0.386
Age at onset, years	58 (10)	58 (10)	NA	63 (10)	63 (11)	63 (9)	–	0.281
MMSE	22.6 (9.2)	17.5 (9.5)	29.3 (0.6)	22.4 (5.8)	23.0 (6.3)	21.5 (5.1)	>28	0.581
CDR SOB	2.8 (5.0)	8.9 (5.2)	0.0 (0.0)	2.8 (4.1)	5.7 (4.9)	3.3 (3.2)	0.0 (0.0)	0.256
CDR global	0.5 (0.8)	1.5 (0.7)	0.0 (0.0)	0.6 (0.7)	1.1 (0.7)	0.8 (0.5)	0.0 (0.0)	0.218
Progranulin DBS^capillary^, ng/mL	2.38 (1.0)	2.36 (0.8)	2.39 (1.2)	4.37 (0.68)	4.2 (0.83)	4.1 (0.46)	4.2 (0.63)	<0.001
Progranulin plasma, ng/mL	87.1 (24.0)	84.2 (35.0)	88.4 (19.3)	243 (59.3)	239 (72.65)	245 (46.3)	241 (55.1)	<0.001

*Note*: Results are expressed as mean (SD) unless otherwise specified.

Abbreviations: AD, Alzheimer's disease; CDR, Clinical Dementia Rating scale (CDR plus NACC was considered in FTD cases); FTD, frontotemporal dementia; *GRN*, *granulin*; HC, healthy control; MMSE, Mini‐Mental State Examination; *n*, number; SOB, sum of boxes.

^a^ Chi‐squared test.

* *p* values: *GRN* mutation carriers (all) versus *GRN* non‐mutation carriers (all), two‐sided *t* test unless otherwise specified.

### 2.2 Collection of plasma and DBS^capillary^ for progranulin dosage

All participants underwent venous plasma and DBS^capillary^ collection. Venous plasma samples were collected in Ethylenediaminetetraacetic acid (EDTA)‐treated tubes, centrifuged (1000 g for 15 min), and stored at −30°C until further analysis. Simultaneously, finger‐stick blood collection was carried out using a single‐use lancet (Accu‐Check Safe‐T‐Pro Plus, Roche, 23G), and 50 µL transferred to a dried blood spot (DBS) card (Capitainer B50, Capitainer AB, Solna, Sweden), stored at room temperature, and shipped without temperature control or cooling within 10 days (1‐10 days). To test sample stability, in a subset (*n* = 21), two DBS^capillary^ cards were collected, one sent overnight and the other after 8 days (range: 7 to 13 days).

### 2.3 Progranulin analysis

Before analysis, one filter from a single Capitainer B50 card containing dried capillary blood was transferred to a spin bucket with fritted bottom (Spin‐X CLS9301, Corning Costar, Cambridge, MA, USA) inside a 1.5‐mL Eppendorf tube. Samples were incubated shaking with 110 µL assay diluent at 37°C and 500 rpm for 30 min. After incubation, the tube was centrifuged at 20°C and 14,000 g for 5 min. After spinning, the eluate was diluted with 500 µL assay diluent and immediately analyzed with progranulin human ELISA kits according to the manufacturers' instructions (Adipogen Life Science, Füllinsdorf, Switzerland; Lot No. K869230). Corresponding EDTA plasma samples were diluted 1:200 with assay diluent. Paired DBS^capillary^ and EDTA samples were analyzed in singlicates in the same experiment. DBS^capillary^ progranulin freeze–thaw cycle stability was determined by repeating the measurement on a subset of the DBS^capillary^ samples (*n* = 10) after one freeze–thaw cycle. Progranulin analyses were run at the Clinical Neurochemistry Laboratory of the University of Gothenburg (Sweden); the interassay coefficient of variation (CV) was 14%, and the intraassay CV was 9%.

**RESEARCH IN CONTEXT**

1. **Systematic review**: The authors performed a literature search using PubMed to identify relevant publications. Recent pilot evidence suggests that extraction methods and analysis of dried blood spots from finger‐stick blood collection (DBS^capillary^) represent an exciting avenue of research. This novel technique might lead to an unprecedented advancement in the design of clinical trials testing disease‐modifying therapies, such as in frontotemporal dementia due to *progranulin (GRN)* mutations.
2. **Interpretation**: Our findings indicated that progranulin levels measured using DBS^capillary^ were significantly reduced in *GRN* mutation carriers compared to non‐mutation carriers and strongly correlated with those from venous blood collection.
3. **Future directions**: DBS^capillary^ might allow repeatedly measuring progranulin levels in a remote and unsupervised setting, circumventing the limitations of traditional venous blood collection. This technique might be used to assess the biological efficacy of disease‐modifying therapies in clinical trials or as a first‐level screening for *GRN* mutations in primary settings.

### 2.4 Statistical analysis

Mean progranulin concentrations in EDTA plasma and DBS^capillary^ were compared using Mann–Whitney U tests. Associations between progranulin levels in EDTA plasma and DBS^capillary^ samples were assessed using Spearman's correlation coefficient. The performance of EDTA plasma and DBS^capillary^ progranulin in identifying *GRN* mutation carriers was done using the receiver operating characteristic curve (ROC) area under the curve (AUC). To evaluate sample stability, two‐sided *t* tests were performed to check for group differences. All statistical analyses were performed in GraphPad Prism 9 for Mac. Significance levels were set at *p *< 0.05.[Table alz14259-tbl-0001]

## 3 RESULTS

A total of 16 *GRN* mutation carriers (mean [SD] age, 55 [13] years; *n* [%] 12 females [75%]) and 44 non‐mutation carriers (mean [SD] age, 64 [11] years; *n* [%] 21 females [48%]) were enrolled in the study.

**FIGURE 1:**
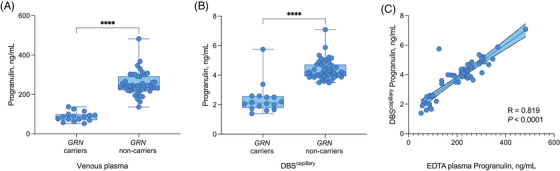
Box and whiskers plot demonstrating levels and differences of progranulin between *GRN* mutation carriers and non‐carriers in (A) Venous Plasma and (B) DBS^capillary^. The correlation between the two methods is shown in (C); displayed are the single data points and the 95% CI. DBS, dried blood spot; EDTA, Ethylenediaminetetraacetic acid; *GRN*, *granulin*. ****p < .0001.

In EDTA plasma, a significant reduction of progranulin levels in *GRN* mutation carriers (median [SD], 87.3 ng/mL [24.1]) compared to non‐mutation carriers (243 ng/mL [59.3]) was observed (*U* = 1; *p *< 0.0001; Figure 1A). Comparatively, DBS^capillary^ progranulin levels were also significantly reduced in *GRN* mutation carriers (median [SD], 2.38 ng/mL [1.0]) compared to non‐mutation carriers (4.37 ng/mL [0.68]; U = 42; *p *< 0.0001; Figure 1B). Progranulin levels were highly correlated between the two methods (*R* = 0.819; *p *< 0.001; Figure 1C). Both EDTA plasma (AUC = 0.99, 95% CI: 0.98 to 1.00) and DBS^capillary^ (AUC = 0.94, 95% CI: 0.83 to 1.00) progranulin levels showed high accuracy in identifying *GRN* mutation carriers. A DBS^capillary^ progranulin cut‐off concentration of 3.44 pg/mL demonstrated a sensitivity of 1.00 and specificity of 0.94. We found that presymptomatic and symptomatic *GRN* mutation carriers could not be distinguished by either collection method (EDTA plasma, *U* = 24, *p *= 0.743; DBS^capillary^, *U* = 24, *p *= 0.721).

**FIGURE 2:**
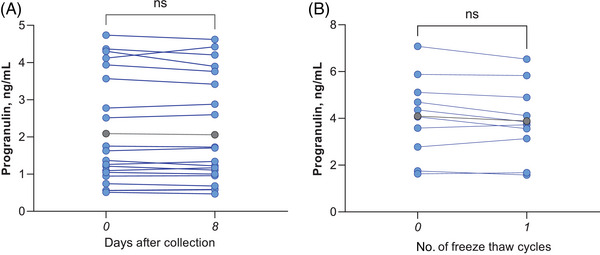
Stability and test retest of progranulin from DBS^capillary^ over 8 days (median, range: 7 to 13 days) of storing at (A) room temperature and (B) after one freeze–thaw cycle. Displayed are the individual values before and after storing/freezing in blue; the mean is plotted in gray. DBS, dried blood spot. ns, non‐significant, *p *> 0.05.

Importantly, in a test–retest experiment, high reproducibility of the extraction protocol (*t*(40) = 0.074; *p = *0.941; Figure 2A), high stability of DBS^capillary^ progranulin when stored at room temperature and of the extract after one freeze–thaw cycle was demonstrated (*t*(20) = 0.303; *p = *0.765; Figure 2B).

## 4 DISCUSSION

In this study, we demonstrated that progranulin levels could be reliably quantified from finger‐stick DBS card collection and strongly correlated with paired EDTA plasma. DBS^capillary^ progranulin was able to identify *GRN* mutation carriers with high accuracy. The DBS^capillary^ extraction method was highly reproducible, and progranulin levels were stable on DBS cards and after one freeze cycle of the extract.

The findings of this quantification method for progranulin, if replicated in larger cohorts with a longitudinal design, might have the capacity to affect clinical practice and refine the design of clinical trials in individuals with *GRN* mutations. DBS^capillary^ have the potential to monitor progranulin levels over time in clinical trials, testing disease‐modifying therapies as an outcome of biological efficacy. Indeed, this method overcomes the limitations of standard venous blood collection, including reduced invasiveness and independence from the infrastructure required for venous blood sample processing, such as centrifuges and ultra‐low temperature freezers. DBS^capillary^ can be implemented in a domestic setting, enabling participants to undergo DBS^capillary^ collection repeatedly and autonomously or by caregivers.

Moreover, DBS^capillary^ can be proposed as a first‐level screening test to identify *GRN* mutations upon clinical suspicion, making the diagnostic process simpler and more cost‐effective with an estimated cost ratio of up to 1:6 compared to remote venous sampling. To do this, future studies should continue to test the accuracy of the DBS^capillary^ progranulin and establish cut‐off values by applying this protocol to larger cohorts. In this analysis, one outlier was observed in the DBS^capillary^ data, which remained after repeating the analysis of the frozen DBS^capillary^ extract. As the corresponding EDTA plasma was correctly determined to be low in this *GRN* mutation carrier, this implies an inaccuracy in the DBS^capillary^ extraction protocol of this one sample. Although this did not change the conclusion of our results, further work will need to deduce the commonality of such anomalies.

In conclusion, DBS^capillary^ could represent an innovative clinical tool for the quantification of progranulin levels. Wider implementation and standardization in a simple test could provide a quicker, less invasive, and more cost‐effective option for disease detection and monitoring in FTD.

## AUTHOR CONTRIBUTIONS

Valentina Cantoni, Daniele Altomare, Ilenia Libri, and Maria Sofia Cotelli conducted the clinical trial. Hanna Huber and Lana Grötschel performed the laboratory analysis. Valentina Cantoni, Daniele Altomare, Barbara Borroni, Hanna Huber, and Nicholas J. Ashton performed the statistical analysis. Nicholas J. Ashton and Barbara Borroni supervised the trial and the laboratory analysis. Hanna Huber and Nicholas J. Ashton created and edited the figures. Valentina Cantoni, Daniele Altomare, Barbara Borroni, Hanna Huber, and Nicholas J. Ashton prepared the first draft of the original manuscript, which was subsequently finalized in close collaboration with Laia Montoliu‐Gaya, Francisco Meda, Hlin Kvartsberg, Henrik Zetterberg, and Kaj Blennow. All authors provided substantial content contributions and edited the manuscript. All authors have read and approved the final manuscript.

## CONFLICT OF INTEREST STATEMENT

K.B. has served as a consultant and on advisory boards for Acumen, ALZPath, BioArctic, Biogen, Eisai, Lilly, Moleac Pte. Ltd., Novartis, Ono Pharma, Prothena, Roche Diagnostics, and Siemens Healthineers; has served on data monitoring committees for Julius Clinical and Novartis; has given lectures, produced educational materials, and participated in educational programs for AC Immune, Biogen, Celdara Medical, Eisai, and Roche Diagnostics. K.B. is a co‐founder of Brain Biomarker Solutions in Gothenburg AB (BBS), which is a part of the GU Ventures Incubator Program, outside the work presented in this paper. H.Z. has served on scientific advisory boards and/or as a consultant for Abbvie, Acumen, Alector, Alzinova, ALZPath, Amylyx, Annexon, Apellis, Artery Therapeutics, AZTherapies, Cognito Therapeutics, CogRx, Denali, Eisai, Merry Life, Nervgen, Novo Nordisk, Optoceutics, Passage Bio, Pinteon Therapeutics, Prothena, Red Abbey Labs, reMYND, Roche, Samumed, Siemens Healthineers, Triplet Therapeutics, and Wave; has given lectures in symposia sponsored by Alzecure, Biogen, Cellectricon, Fujirebio, Lilly, Novo Nordisk, and Roche; and is a co‐founder of Brain Biomarker Solutions in Gothenburg AB (BBS), which is a part of the GU Ventures Incubator Program (outside submitted work). B.B. has served on scientific advisory boards and/or as a consultant for Alector, Aviado Bio, UCB, Lilly, Denali, and Wave. N.J.A. has given lectures, produced educational materials, and participated in educational programs for Eli Lilly, BioArtic, and Quanterix. Author disclosures are available in the Supporting Information.

## CONSENT STATEMENT

All participants gave written informed consent according to the Declaration of Helsinki.

## Supporting information



ICMJE Disclosure Form

## Data Availability

Data are not provided in the article because of space limitations and may be shared (anonymized) at the request of any qualified investigator for purposes of replicating procedures and results.
